# Susceptibility of Snails to Infection with Schistosomes is influenced by Temperature and Expression of Heat Shock Proteins

**DOI:** 10.4172/2161-1165.1000189

**Published:** 2015-06-21

**Authors:** Matty Knight, O Elhelu, M Smith, B Haugen, A Miller, N Raghavan, C Wellman, C Cousin, F Dixon, V Mann, G Rinaldi, W Ittiprasert, PJ Brindley

**Affiliations:** 1 University of the District of Columbia, 4200 Connecticut Ave, Washington, D.C. 20008, USA; 2 Schistosomiasis Resource Center, Biomedical Research Institute, 12111 Parklawn Drive, Rockville, USA; 3 George Washington University, Department of Microbiology, School of Medicine and Health Sciences, 2300 Eye Street NW, Washington, D.C, USA

**Keywords:** *Biomphalaria glabrata*, *Schistosoma mansoni*, Resistant BS-90 snails, Susceptibility, Global warming

## Abstract

The freshwater snail, *Biomphalaria glabrata* is the obligate intermediate host for the transmission of the parasitic trematode, *Schistosoma mansoni* the causative agent of the chronic debilitating neglected tropical disease, schistosomiasis. We showed previously that in juvenile snails, early and significant induction of stress manifested by the expression of stress proteins, Hsp 70, Hsp 90 and reverse transcriptase (RT) of the non- LTR retrotransposon, nimbus, is a characteristic feature of juvenile susceptible NMRI but not resistant BS-90 snails. These latter, however, could be rendered susceptible after mild heat shock at 32°C, revealing that resistance in the BS-90 resistant snail to schistosomes is a temperature dependent trait. Here we tested the hypothesis that maintenance of BS-90 resistant snails at the permissive temperature for several generations affects the resistance phenotype displayed at the non-permissive temperature of 25°C. The progeny of BS-90 snails bred and maintained through several generations (F1 to F4) at 32°C were susceptible to the schistosome infection when returned to room temperature, shedding cercariae at four weeks post-infection. Moreover, the study of expression levels of the heat shock protein (Hsp) 70 protein by ELISA and western blot analysis, showed that this protein is also differentially expressed between susceptible and resistant snails, with susceptible snails expressing more protein than their resistant counterparts after early exposure to wild-type but not to radiation-attenuated miracidia. These data suggested that in the face of global warming, the ability to sustain a reduction in schistosomiasis by using refractory snails as a strategy to block transmission of the disease might prove challenging since non-lethal elevation in temperature, affects snail susceptibility to *S. mansoni*.

## Introduction

Schistosomiasis is a major neglected tropical disease (NTDs) that remains refractory to control. It is a chronic debilitating disease of poverty that persists in over 75 countries of the tropics and subtropics, causing at least 200,000 deaths a year [1–5]. Its recent emergence in Corsica (France) confirms its spread from Africa to regions at higher latitudes [6–8]. Freshwater gastropod snails are obligate hosts for the development of asexual stages of the trematode parasite that causes schistosomiasis. Lately, Mass Drug Administration (MDA) alone (with praziquantel) to control schistosomiasis has shown underwhelming impact in curtailing transmission [3,9–11]. WHO has projected 2050 as the year for global elimination of schistosomiasis [12]. However, without a vaccine to prevent the disease and understanding that MDA alone will fail to eradicate schistosomiasis, alternative control approaches are desperately needed. One control approach first proposed in the 1950s by Hubbendick [13,14], aims to introduce resistant snails into endemic foci to replace the population of resident susceptible snails as a form of biological control to block transmission. Interestingly, as proof of principle, Jordan working on the Island of St Lucia in the 1970s, used a non-compatible snail, *Biomphalaria straminae*, to block transmission of *S. mansoni* in the part of the island where this form of control was implemented. This study and others showed that snails resistant to *S. mansoni* indeed provided a means to break the schistosome life cycle, something that had been previously accomplished by using molluscicides [15]. Though the application of molluscicides has been effective in curtailing schistosomiasis, there is the risk of pollution and destruction of fragile ecosystems. In addition, the repeated cost of applying these molluscicides is economically non-sustainable. Therefore, with the rationale that a snail vector control strategy would be the most environmentally friendly and cost effective method for disruption and spread of schitosomiasis in the long-term, a molecular approach was undertaken two decades ago by several investigators to identify pathway(s) in the snail/schistosome interaction that underscore resistance/susceptibility of the snail host to schistosome infection [15,16]. Resistant (BS-90) and susceptible (NMRI) *Biomphalaria glabrata* snail strains were exposed to *Schistosoma mansoni*, and differences in early gene expression investigated. Several genes were found to be differentially expressed between these two strains [17]. For instance, among others, stress genes encoding Hsp 70 and Hsp 90 were upregulated in NMRI (susceptible) compared to the BS-90 resistant snails after *S. mansoni* infection. BS-90 snails, resistant at room temperature, when subjected to non-lethal heat shock at 32°C prior to exposure became susceptible to the infection. Moreover, the treatment of susceptible NMRI snails with the Hsp 90 inhibitor, geldenamycin, rendered these snails resistant [18].

Heat shock proteins (Hsps) are evolutionarily highly conserved molecular chaperones that function to protect the cell during stressful conditions. Hsps are actively synthesized in response to any cellular stress from heat shock, infection, or trauma. Their ability to bind to mis-folded and newly synthesized proteins during stress prevents protein aggregation, and therefore, cell-death. Although Hsps are intracellular proteins, they also are released in exosomes but possibly also released because of cell death can activate the innate immune response [19,20]. Herein we tested the hypothesis that maintaining BS-90 resistant snails at the permissive temperature of 32°C for several generations will affect their normal resistance phenotype at the non-permissive temperature of 25°C. Progeny of BS-90 snails bred and maintained through several generations (F1 to F4) at 32°C when returned to 25°C were no longer resistant, shedding cercariae at four weeks post-exposure at 25°C. Additionally, 90 the expression of Hsp 70 protein by ELISA and western blot analysis was higher in susceptible snails than in their resistant counterparts after the exposure to normal but not to irradiated-attenuated miracidia.

## Materials and Methods

### Maintenance of snails and exposure of snails to schistosomes

Laboratory stocks of *B. glabrata* that are either susceptible (NMRI stock, 100 to 90% susceptible) or resistant (BS-90, 100% at 25°C) were maintained in fresh de-chlorinated tap water and fed ad libitum on romaine lettuce. Snails were exposed either as juveniles (between 3 to 4 mm in diameter) or as young adults (between 6 to 7 mm in diameter). Snails were exposed to the NMRI strain of *S. mansoni* in 12-well microtiter plates using either eight miracidia/juvenile snail or 12 miracidia/young adult snail. Mice infected with *S. mansoni* were euthanized by lethal injection seven weeks post-exposure. Livers from mice were homogenized in a blender to collect eggs that were hatched in fresh water under a lamp to attract the free-swimming miracidia. Radiation-attenuated miracidia were prepared as previously described [21].

### BS-90 breeding at 32°C, exposure of snails, shedding of schistosome cercariae

**Figure 1:**
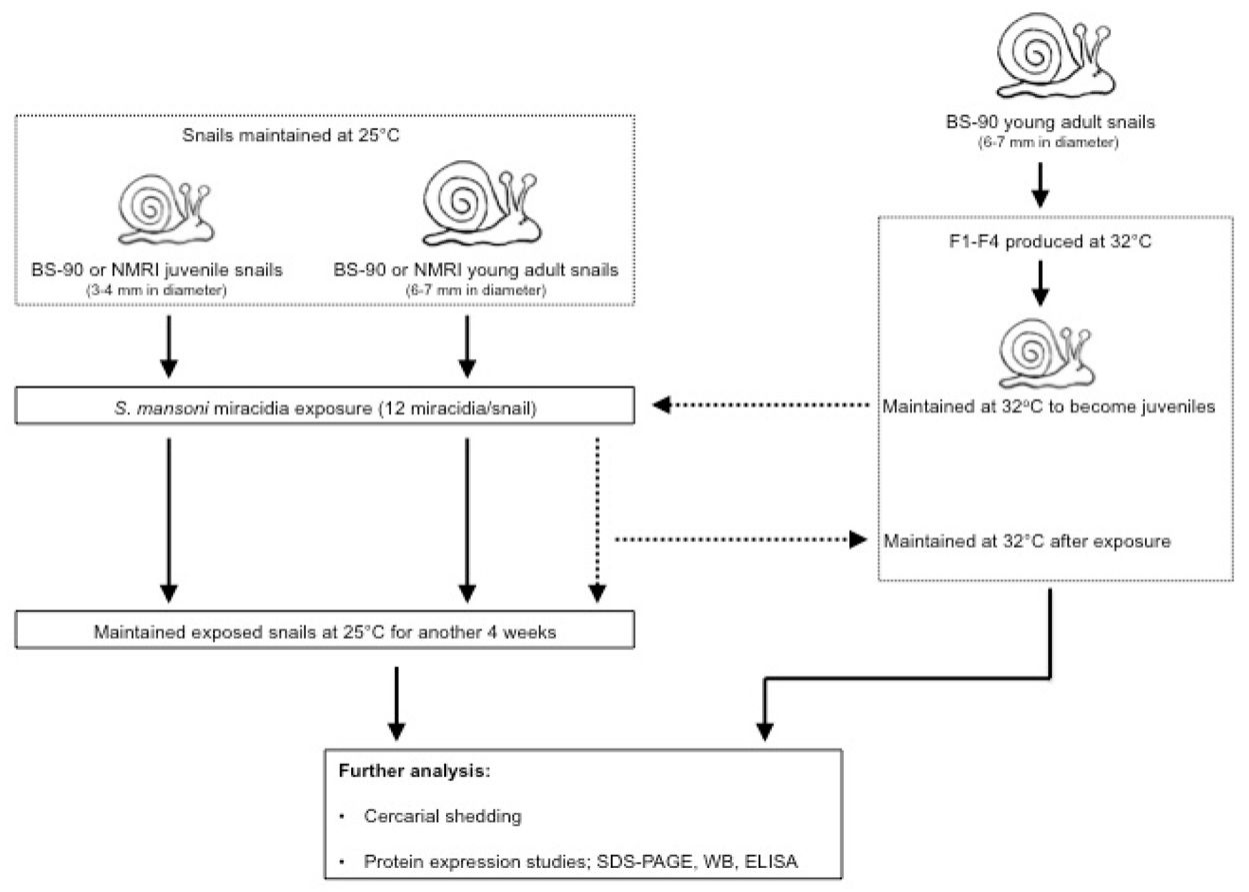
A schematic illustration of the experimental design. Note that BS-90 offspring produced at 32°C were exposed to schistosome miracidia at 25°C.

A schematic workflow describing the experimental design utilized herein is shown in Figure 1. The resistant 25°C young adult BS-90 snails initially introduced to 32°C were maintained as individuals in fresh water in beakers (100 ml) that were kept in an incubator at 32°C. The temperature inside the incubator was monitored daily to maintain 32°C for the duration of the experiment. Dead snails and old lettuce leaves were removed immediately from the beakers. The BS-90 snails at this temperature were fed as above on romaine lettuce. Snails were cleaned each week using pre-warmed (32°C) water. Egg clutches first produced from the individual parent (F^o^) snails introduced from 25°C to 32°C were discarded. Thereafter, snails were combined into a single beaker (500 ml) and the egg clutches from these snails (produced at 32°C) were collected and their progeny (F1 to F4) cultivated into juveniles before exposure to miracidia at 25°C as described above. After exposure, snails were kept at room temperature for two hours and used for soluble protein isolation either immediately or 116 after they were frozen at −80°C. Some of the exposed BS-90 (32°C) snails remained at room temperature or were returned to 32°C. The room temperature and 32°C maintained snails were monitored for mortality and cercarial shedding at four weeks after exposure to miracidia. All snail exposures were done alongside controls that used similarly-sized susceptible NMRI snails, as well as resistant BS-90 room temperature maintained snails that were exposed to the same number and batch of miracidia. Results were compared to normal unexposed snails (NMRI, BS-90 room temperature, and BS-90 32°C).

### Soluble protein extract

Soluble protein extract was prepared from either freshly dissected or frozen tissue (head foot) samples by homogenizing in 1X phosphate buffered saline (PBS, pH 7.4) on ice using a pestle and mortar followed by centrifugation at 10,000g for 20 minutes at 4°C. The supernatant was retained, protease inhibitor cocktail (Calbiochem, San Diego, CA, USA) added, and protein concentration estimated using the Bradford assay [22]. Electrophoresis was performed using 25 μg of protein sample after the addition of an equal volume of reducing 2X SDS-PAGE sample buffer (0.125 M Tris-HCl, pH 6.8, 4% SDS, 20% glycerol, 2% *β*-mercaptoethanol, 0.02% bromophenol blue) on 10% separating gels according to the method of Laemmli (1970) [23]. After electrophoresis, gels were stained in Coomassie blue and destained with acetic acid/methanol buffer (10% v/v). Gels for western blotting were processed as described below.

### Expression of the snail BgHsp 70 in *E. Coli*: purification 136 and preparation of antibody against the recombinant Hsp 70 protein

The full length BgHsp 70 Open Reading Frame (ORF) of 1908 bp was amplified by PCR and expressed as a His-tagged fusion protein using the method of Ghosh et al. [24]. Briefly, the BgHsp 70 ORF was amplified using a 5' primer (hsp 70-2exp5’):

5’-CGC GGATCC T ATGTCAGGACGAAATAAGGCTCCC-3’ (34-mer)

overhang BamHI spacer - HSP 70 5’ ORF (24 bp)

Containing a recognition site for BamH1, a 5’overhang to ensure proper cutting by the restriction enzyme and a spacer (T) to keep the ORF in frame and a 3' primer (hsp 70-2exp3’):

5’-CGG GAATTC TCA ATCTACTTCTTCAACTGTTGGGCC-3’ (36-mer)

overhang EcoRI stop - HSP 70 3’ORF (24 bp)

Containing a recognition site for EcoR1, a 5’overhang to ensure proper cutting by the restriction enzyme and a stop codon. A three step-cycle PCR program of 94°C for 1 min, 65°C for 1 min and 72°C for 1.5 min was employed for 30 cycles. The gel-purified PCR product of 1930 bp was digested with BamH l and EcoR1 and ligated into the expression vector pRSET B (Invitrogen, Carlsbad, CA) in frame. The recombinant plasmid was used to transform *E. coli* (DH10B). In order to express the recombinant protein from the BgHsp 70-pRSETB constructs, purified recombinant DNA from the transformants propagated in *E. coli* DH10B were checked for the correct size and orientation (in-frame) and verified by PCR prior to transformation into *E. coli* B strain BL21 (DE3) pLYsS (Invitrogen, Carlsbad, CA). Protein expression in the transformants containing the BgHsp 70 insert was induced with 0.4 mM IPTG for 2 h at 37°C according to manufacturer’s instructions (Invitrogen, Carlsbad, CA). The expressed BgHsp 70-His-tagged fusion protein was purified using a nickel-NT agarose 158 column. For elution, buffer contained varying concentrations of imidazole (60 mM-500 mM) according to the manufacturer's recommendation (Novagen/EMD Biosciences, Madison, WI). The recombinant protein, eluted in 200 mM imidazole, was dialyzed against Tris-buffered saline (TBS, pH 8.0), and quantitated by the bicinchoninic protein assay (Pierce, Rockford, IL). Antibodies were produced in six Swiss Webster mice (Charles River Laboratories Inc., Wilmington, MA) using purified recombinant BgHsp 70 protein. Approximately 50 μl of blood was obtained from each mouse (pre-immune) by collecting blood from the tail vein by a needle-prick procedure. For primary immunizations, 15 μg of purified BgHsp 70 protein was mixed with 40 mg/ml alum (1:1) in a total volume of 300 μl and injected subcutaneously in the right flank. At three-week intervals, two subsequent boosts (300 μl volume) were administered subcutaneously in separate sites, with 5 μg of the purified protein/alum mixture (as above). Following collection of blood, antibody titers were determined by western blotting (see below) throughout the course of immunization, prior to each booster immunization, and at three weeks after the final boost. Mice were then euthanized by an IACUC approved CO2 inhalation procedure, and blood was collected by cardiac puncture. The Biomedical Research Institute’s (BRI) animal care program complies with the guidelines adopted by the Office of Laboratory Animal Welfare (OLAW) and maintains an OLAW Assurance (AALAC license number 000779). No experimental animals were used unnecessarily and care was taken to avoid distress. All animal procedures were conducted by protocols approved by the BRI’s animal care and use committee (IACUC protocol number 09-03).

### Enzyme linked 180 immunoabsorbent assay (ELISA)

Soluble protein extracts were diluted to final concentrations of 5 μg, 10 μg, 15 μg, and 20 μg in 100 μl of 1XPBS (pH 7.4) in a 96-well microtiter plate (flat bottom, Becton Dickinson, Franklin Lakes, New Jersey, USA). Blocking buffer (Leinco Prod. No. B396, Leinco Technologies, St. Louis, Missouri, USA) was added (200 μl) to the wells. Plates were sealed and incubated overnight at 4°C before washing 3X in 1XPBS/Tween (0.05% v/v) Primary antibody (mouse anti-Hsp 70 serum diluted at 1:1000 in 1X PBS pH 7.4) was added for one h at 37°C and washed 3X with 1XPBS/Tween. Secondary antibody (goat anti-mouse HRP conjugate diluted 1:1000 in 1X PBS pH 7.4) in 100 μl was added to each well and plates were incubated for one h at 37°C. Plates were washed 3X with 1X PBS/Tween before developing using substrate (ABTS peroxidase substrate Kirkegaard and Perry Laboratories, Gaithersurg, Maryland, USA). Absorbent readings were taken at 450 nm in an iMark microplate reader (BioRad,) previously calibrated against known concentrations of a protein standard (Bovine Serum Albumen fraction V). ELISA analysis of this standard and snail protein extracts was also performed with controls that used pre-immune mouse serum as primary antibody in the wells. Background readings were also taken with wells that contained either snail protein extract alone (antigen only) or antiserum only as controls.

### Western blotting

Soluble protein extracts, from individual BS-90 and NMRI, either juvenile or adult snails, were homogenized in sterile 1XPBS, pH 7.5 on ice using a mechanized Kontes pestle (VWR, West Chester, PA). Soluble protein was prepared as above. The protein concentration in the supernatant was determined by the BCA method [25]. Soluble extracts (50 μg) were resolved under reducing conditions by SDS-PAGE as above. Western blot (Towbin) was performed by transferring resolved protein onto a nitrocellulose membrane 203 using a semi-dry blotter in 0.192 M glycine, 0.025 M Tris pH 8.3, 0.0013 M SDS and 10–20% methanol [26]. After transfer for 1 h at 10–15 V/200-300 mA, proteins transferred to the membrane were visualized using 0.4% Ponceau S stain in 2% trichloroacetic acid prior to blocking and antibody addition. The membrane was then blocked using blocking buffer (TBS, 0.1% Tween-20, 0.05% Triton X-100 and 3% Carnation milk powder) for 2 h at room temperature, followed by incubation with mouse anti-BgHsp 70 antibodies (1:1000 dilution) in blocking buffer overnight at 4°C [27]. The secondary antibody was a 1:1000 dilution of goat anti-mouse IgG Fc conjugated with horse radish peroxidase (SignaGen, Rockville, MD USA) in blocking buffer. Following three washes in TBS, 0.1% Tween-20, 0.05% Triton X-100, the antigen-antibody complexes were visualized by chemiluminescence using a kit (SigmaGen, Rockville, MD, USA).

## Results

### Breeding BS-90 resistant snails at 32°C renders them susceptible to schistosomes at 25°C

**Figure 2:**
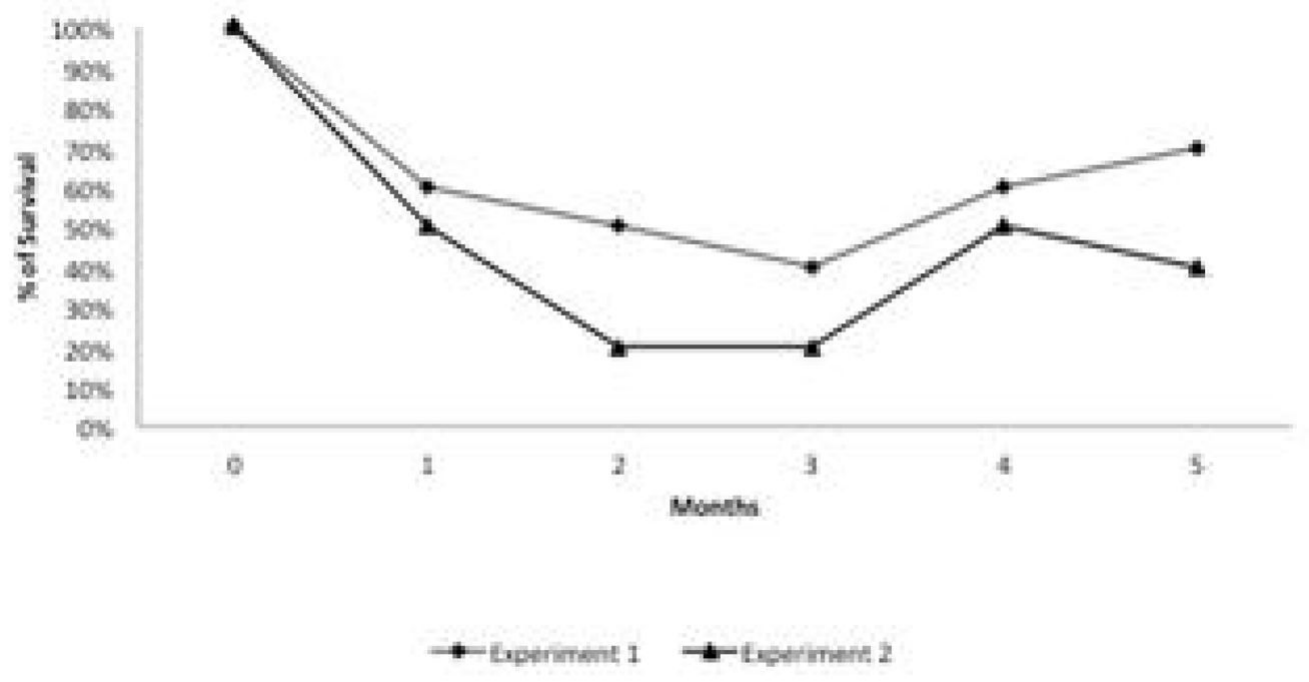
The percentage of BS-90 snails surviving after incubation over time (months) at 32°C. Note the rapid decline in mortality occurred within the first month of introducing the snails to 32°C.

**Figure 3:**
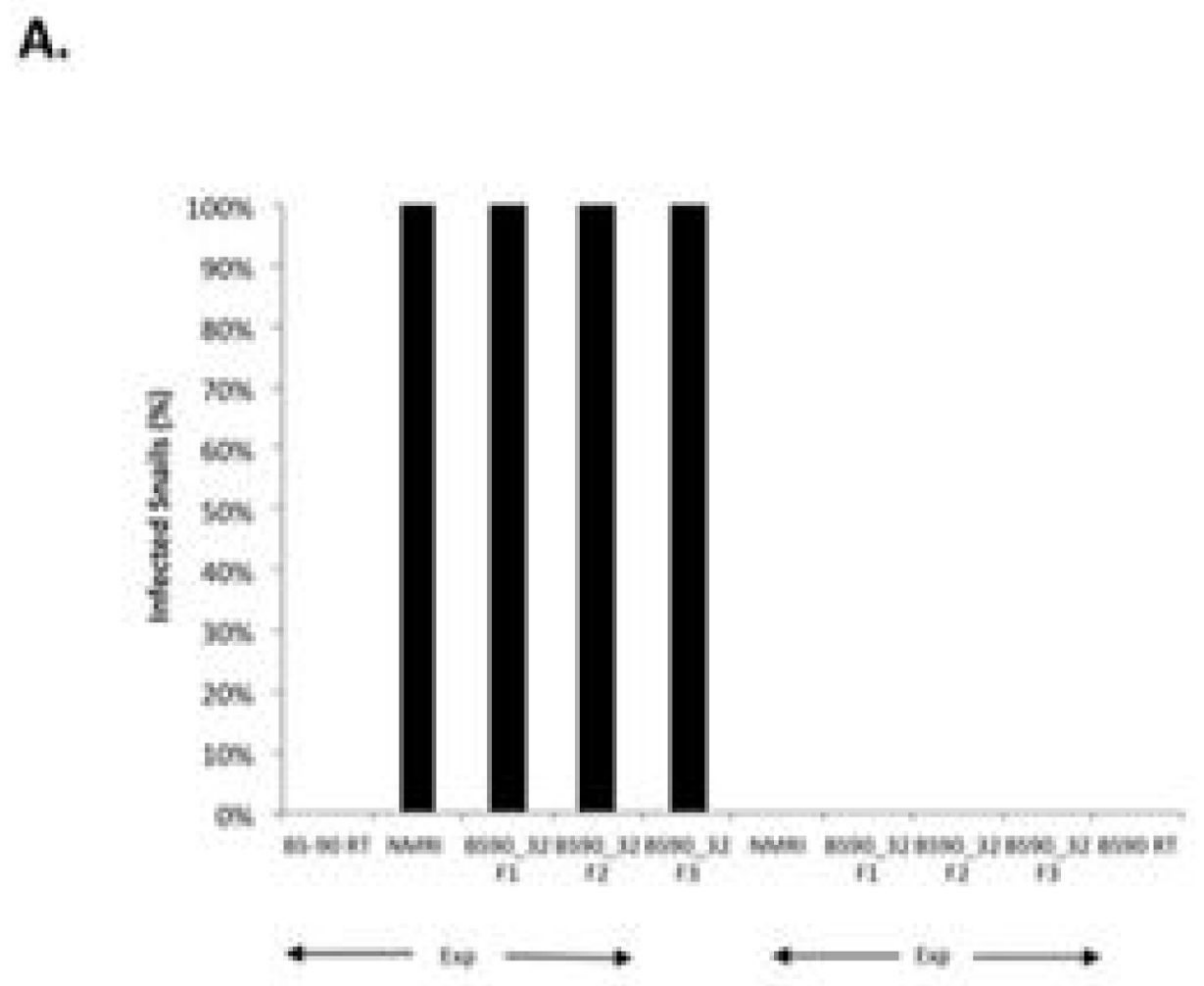

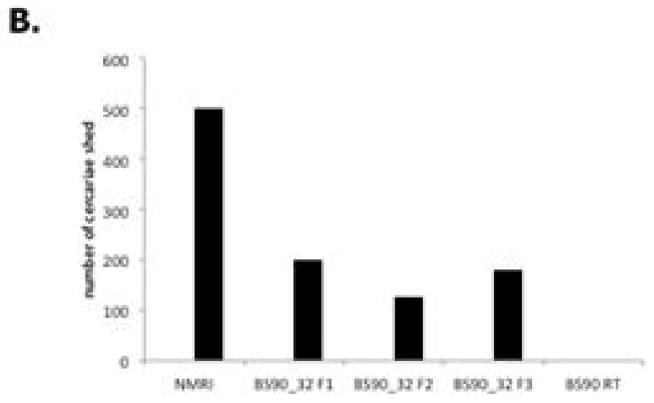
Figure 3A: Histograms showing percentage of susceptible NMRI, BS-90_32 Progeny, F1, F2 and F3 shedding cercaria after 4 weeks post-exposure to *S. mansoni* miracidia. Note that the non-infected control (unexp) BS-90 room temperature (RT) resident snail, exposed (Exp) in parallel to the same number of miracidia, remained negative for infection. Figure 3B: Histograms showing the number of cercariae shed from susceptible NMRI and BS-90_32 F1, F2 and F3 progeny snails. Note that the non-infected control (unexp) room temperature (RT) resident BS-90 exposed in parallel to the same number of miracidia remained negative for infection.

To determine whether breeding resistant BS-90 at the higher temperature of 32°C affected the susceptibility phenotype of their progeny snails produced at this elevated temperature, even after exposure to *S. mansoni* and subsequent maintenance (post-exposure) at room temperature, BS-90 snails were maintained at 32°C, and allowed to produce egg clutches as described in Materials and Methods. As shown in Figure 2, the mortality rate of snails, within a month of being at 32°C was high (results from two separate experiments): with only 50 to 60% of the snails surviving during this time period. Although survival rates were variable between experiments, we observed by the third month of being at the elevated temperature, that survival improved and snails began to produce egg clutches. In both experiments we found that snails kept individually at 32°C failed to produce egg clutches, and therefore we housed snails in a single container to allow them to cross-fertilize. To ensure that only progeny produced at 32°C were investigated, egg clutches produced by the individual snails when they were first introduced at the higher temperature were discarded. All parasite exposures of 32°C maintained BS-90 (BS- 90_32°C) was undertaken at 25°C. These schistosome-exposed BS-90_32°C progeny (F1 to F4) snails were either maintained at room temperature or returned after exposure to 32°C to monitor for shedding of cercariae. In addition, BS-90_32°C progeny after two hours’ exposure were frozen and used for protein extraction. As shown in Figure 3A, all the 32°C maintained progeny BS-90 (100% from both experiments) even as early as the first generation (F1) or later (F4, data not shown) were found to be positive at four weeks post-exposure, within the same time frame as the highly susceptible NMRI snail. Intriguingly, all the BS-90_32°C progeny that were returned to 32°C after exposure failed to survive (data not shown). Although all the exposed BS-90_32progeny (F1 to F3) were found to be positive for infection as shown in Figure 3B, they however, shed fewer cercariae than exposed susceptible NMRI snails. Room temperature (25°C) - BS-90 snails that were exposed at the same time as the 32°C progeny snails failed to shed cercaria and, as anticipated, were resistant to *S. mansoni* (Figures 3A and 3B).

### Susceptible and resistant juvenile snails display different levels of Hsp 70 in response to either heat shock or infection with *S. mansoni*

**Figure 4:**
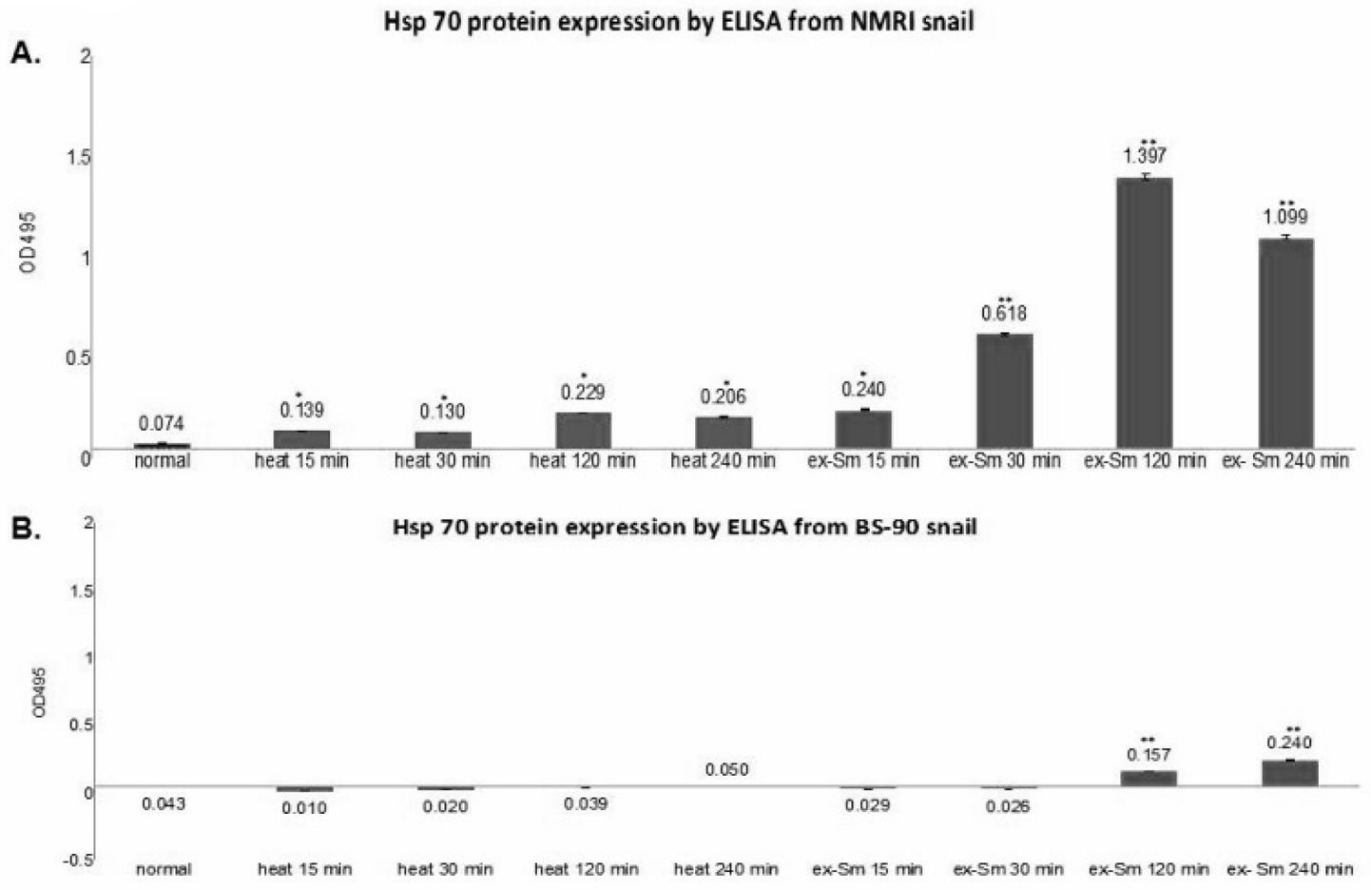
A) Histograms showing absorbance at OD495 of susceptible NMRI snail soluble protein extracts in an ELISA assay using mouse antiserum raised against the purified snail recombinant Hsp 70 protein expressed in *E. coli.* Protein extracts were prepared from snails, either after heat shock at 32°C or following exposure for different time points to miracidia as described in Materials and Methods. Note that more Hsp 70 expression was observed following infection than by heat shock in the susceptible NMRI strain. Results were from three biological replicates. B) Histograms showing absorbance at OD495 of resistant BS-90 snail soluble protein extracts in an ELISA assay using mouse antiserum rose against the purified snail recombinant Hsp 70 protein expressed in *E. coli*. Protein extracts were prepared from snails, either after heat shock at 32°C or following exposure to miracidia for different time points as described in Materials and Methods. Note that more Hsp-70 expression was observed following infection than from heat shock. Results were from three biological replicates.

Resistant BS-90 and susceptible NMRI snails respond differently in the temporal kinetics and levels of Hsp 70 mRNA induced after either heat shock at 32°C or after early exposure to miracidia [21]. Here we quantified the Hsp 70 protein by ELISA in protein extracted at different time points after exposure to schistosome miracidia or heat shock for 15, 30, 120, or 240 minutes from NMRI susceptible (Figure 4A) and BS-90 resistant (Figure 4B) snails.

As shown in Figure 4 either heat shock or *S. mansoni* infection induced enhanced expression of Hsp 70 in the susceptible NMRI (Figure 4A) than the resistant BS-90 (Figure 4B) at all-time points. Compared to the susceptible snail where Hsp 70 protein expression was observed early after schistosome infection (30 min), Hsp 70 protein induction was low and slow (120 min) in the resistant snail. Interestingly, in the susceptible NMRI snail higher levels of Hsp 70 protein were detected following *S. mansoni* infection than after the heat shock. Analysis using ELISA of protein extracts from these groups of snails with the pre-immune antiserum, as control, detected no absorbance (data not shown). Similarly, no signal was detected in controls with primary antibody alone.

### Western blots revealed differences in expression of Hsp 70 between 258 susceptible and resistant juvenile and adult snails in response to either heat shock or exposure to schistosomes

**Figure 5:**
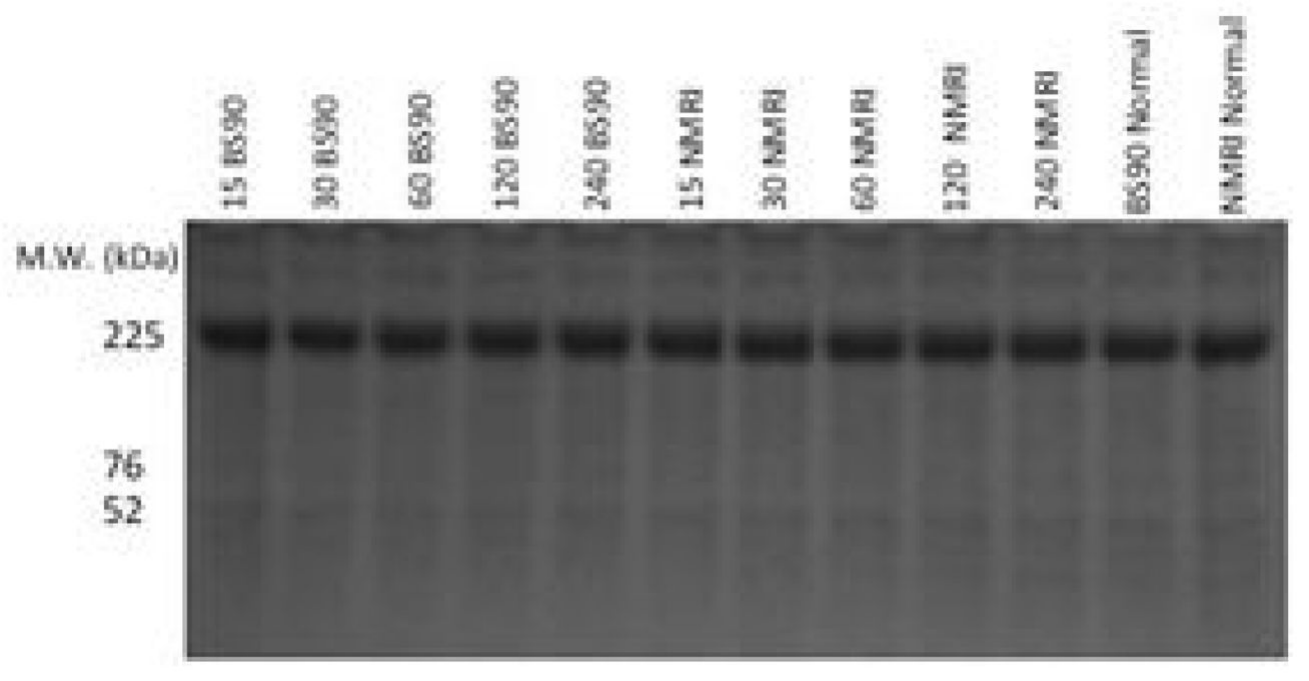

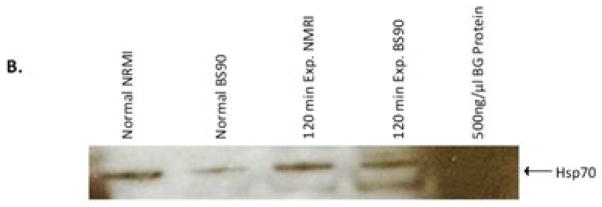
Figure 5A: Reduced SDS-PAGE Coomassie stained gel, showing lanes that were loaded with equal amounts of soluble protein extracts prepared from the exposed snails (NMRI and BS-90) and normal unexposed (control) snails used in this study. The same concentrations of protein extract as described in Materials and Methods was utilized for all western blotting analysis. Note the uniform loading of protein in each lane. The same amount of protein isolated from the heat- shocked (32°C) snails was also utilized for Western blot analysis. Figure 5B: Western blot analysis of protein extracts from normal, 120 minute exposed NMRI and BS-90 snails, and the snail recombinant Hsp 70 protein (positive control). The immunoreactive bands shows the specificity of the antiserum recognizing the Hsp70 protein in extracts from exposed snails and the intense recognition of the purified recombinant protein utilized to raise this antiserum.

To further investigate differences in levels of Hsp 70 protein in BS-90 resistant and NMRI susceptible snails in response to either heat shock or exposure to *S. mansoni* miracidia, we analyzed protein extracts using immunoblots. Extracts from both juvenile and adult snails were examined in order to evaluate if age of the snail is a factor in the stress induction post-infection, a finding that we had observed in juveniles [17]. Protein extracts utilized for western blot analysis were beforehand, stained with Coomassie blue to ensure there was uniform concentration of protein samples loaded (Figure 5A).

In addition, the specificity of the anti-snail Hsp 70 serum was investigated by western blot analysis using protein extracts from normal, 120 min exposed NMRI and BS-90 snails, and positive control *E. coli* BgHsp 70 purified recombinant protein (Figure 5B).

**Figure 6:**
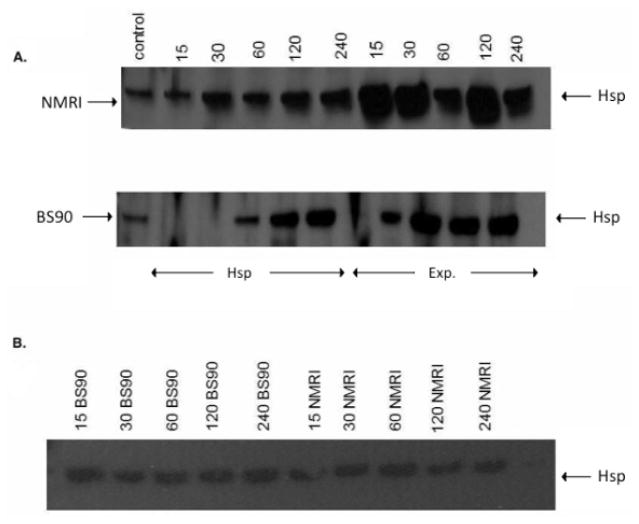

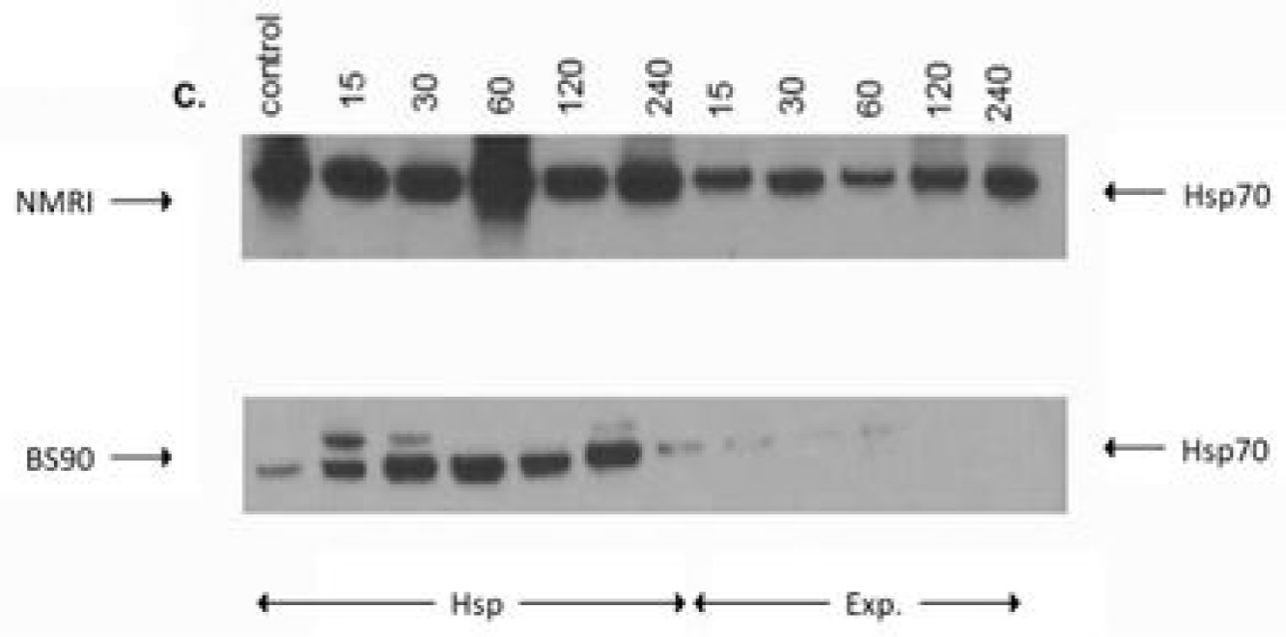
A) Western blot analysis of soluble protein extracts prepared from juvenile susceptible (NMRI) and resistant (BS-90) snails after either heat shock (32°C) or following exposure. for different time points to normal miracidia using anti-Hsp 70 serum as described in Materials and Methods. Note that there is significant expression of Hsp 70 at early time points after normal schistosome exposure in the susceptible NMRI compared to the expression of this protein in the resistant BS-90 snail. Arrows are used to indicate the lanes loaded with protein samples from heat shocked (Hsp) vs. schistosome exposed (Exp) snails. B) Western blot analysis of soluble protein extracts prepared from juvenile susceptible (NMRI) and resistant (BS-90) snails after either heat shock (32°C) or following exposure for different time points to radiation-attenuated miracidia using anti-Hsp 70 serum as described in Materials and Methods. Note expression of Hsp 70 after attenuated parasite exposure in both susceptible NMRI compared to the resistant BS-90 snail remains the same at all-time points examined. Figure 6C: Western blot analysis of soluble protein extracts prepared from adult susceptible (NMRI) and resistant (BS-90) snails after either heat shock (32°C) or following exposure for different time points to normal miracidia using anti-Hsp 70 serum as described in Materials and Methods. Note expression of Hsp 70 after heat shock in susceptible adult NMRI is significant at all-time points when compared to expression of this protein in resistant adult BS-90 snails. Hsp 70 protein expression in adult BS-90 snails, post- exposure at all-time points was negligible. Arrows are used to indicate the lanes loaded with protein samples from heat shocked (Hsp) vs. schistosome exposed (Exp) snails.

As shown in Figure 5B, the anti-snail Hsp 70 serum recognized immunoreactive 70 KDa bands in the snail extracts and also intense recognition of the purified Hsp70 recombinant protein utilized to raise this antiserum thus indicating the specificity of the antiserum. Protein extracts were also prepared from susceptible (NMRI) and resistant (BS-90) juvenile snails at 0, 15, 30, 60 120, and 240 minutes, either after heat shock or after exposure to wild-type (Figure 6A) or radiation-attenuated (Figure 6B) miracidia.

Figure 6C shows Western blot employing adult snail protein extracts from NMRI and BS-90 snails isolated either after heat shock or after exposure to wild-type miracidia. As shown in Figure 6A, Hsp 70 protein was significantly up-regulated in the susceptible NMRI juvenile snail compared to the resistant BS-90 juvenile snails following either heat shock or exposure to these miracidia, e.g., at all-time points post exposure of NMRI susceptible snail to normal miracidia, significant expression of Hsp 70 protein was seen in this snail compared to its resistant (BS-90) counterpart.

Since we had shown previously that radiation-attenuated miracidia, unlike wild-type schistosomes, failed to induce stress, including the Hsp 70 encoding transcript [17], here we were interested to further investigate this outcome at the protein level. Western blot analysis of protein extracts from susceptible and resistant snails exposed for different time periods (15, 30, 60, 120, 240) to attenuated miracidia showed there was no increase in Hsp 70 expression in the snails after exposure to radiation-attenuated miracidia (Figure 6B).

As expected, Hsp 70 was upregulated in susceptible NMRI adult snails in comparison to resistant BS-90 adult snails (Figure 6C). However, whereas elevated expression of Hsp 70 was observed in susceptible juvenile snails responding to *S. mansoni* exposure compared to heat shock, adult snails exhibited less expression of this protein in snails after exposure to the parasite than after heat shock. In fact, in the adult resistant BS-90 snail, western blot analysis of protein extracts after infection showed negligible Hsp 70 protein expression.

## Discussion

Previously we had reported that susceptibility of the intermediate snail host, *B. glabrata* to *S. mansoni* infection, is influenced by elevated temperature. Moreover, we also showed that an early induction of stress was a characteristic feature of susceptible but not resistant snails. Therefore, treatment of the susceptible NMRI snail with the Hsp 90 inhibitor, geldenamycin, rendered this snail non-susceptible to *S. mansoni* [18,28]. In this follow up study, we have shown that breeding the resistant BS-90 snail at 32°C rendered all progeny (F1 and F4) produced in this warmer environment susceptible to *S. mansoni* after exposure at 25°C. The BS-90 snail are normally resistant at ambient temperature and have been considered for the past two decades as the representative resistant snail strain that is used in most molecular studies aiming to identify genes that underscore the infection refractory phenotype [18].

The BS-90 stock is a wild type pigmented snail that was identified in Salvadore, Brazil by Correa and Paraense [29]. At room temperature (25°C), this snail is refractory at all ages (juvenile and adult) to both new and old world *S. mansoni*, reliably destroying the invading miracidia by an active innate defense mechanism where hemocytes and plasma factors quickly kill the larval schistosome [30–32].

For the susceptible NMRI stock used in this study, however, early recruitment of hemocytes that engulf the parasite fails to manifest and the miracidia establish the infection and develop into sporocysts stages that by asexual reproduction produce cercariae that are infectious to the human host. Understanding the complex mechanism(s) behind these diverse outcomes of the schistosome/snail host interaction (notwithstanding the parasites own genetic make-up that also shapes this relationship) has been the driving force for developing new vector-based strategies to control schistosomiasis. In this regard, the anticipation has been to utilize resistant snails either to displace susceptible ones in the field to block transmission of schistosomiasis, or to use resistant snails to elucidate molecular pathways that underlie refractoriness to the schistosome trematode in its intermediate snail host [15,16].

Investigation of differential gene expression between BS-90 and NMRI snails previously revealed the importance of early induction of stress related transcripts (Hsp 70, Hsp 90 and the RT domain of nimbus) and snail susceptibility to schistosome infection [17,21]. By ELISA and Western blot analysis using antiserum raised against the snail recombinant Hsp 70 protein, our results showed that the level of this protein was higher after either heat shock or parasite exposure in the susceptible (NMRI) than in resistant (BS-90) snail. In addition, higher levels of Hsp 70 protein occurred in response to infection than heat shock in the susceptible juvenile snail. These studies were also prompted by discrepancies between our previous studies and other reports that found in contrast to our results, that Hsp 70 mRNA was expressed in resistant but not susceptible snails [33]. This discrepancy remains unexplained, but it is feasible that different t isoforms of Hsp 70 (at both RNA and protein levels) complicate these investigations. The analysis and annotation of the *B. glabrata* genome assembly is ongoing and information of Hsp 70 gene copy number would bring more clarity to this matter (manuscript in preparation).

It is clear from this present study that using a resistant snail such as the BS-90 stock with its inherent degree of variable susceptibility to *S. mansoni* in a vector based strategy to control schistosomiasis in the face of global warming will be difficult. There have been recent reports of climate change exacerbating transmission of schistosomiasis with the disease now re-emerging in parts of Europe where it had been eradicated [6-8]. With the WHO forecast to eliminate schitosomiasis by 2050 [12], no vaccine and only a single effective drug to combat the disease, greater understanding of how the snail adapts and responds to schistosomes in a changing thermal environment is needed. This information is critical since our results suggest that as early as the first generation, these BS-90_32°C snails were 100% susceptible, an indication that the change is rapid. There is increasing interest in the subject of change in the genomics of metazoans and adaptation to temperature [34]. It is possible that epigenetic marks play a role in the variation of the temperature dependent susceptibility of the BS-90 snail stock as seen in this study. In a recent study we have shown that schistosomes indeed have the capability to change the DNA methylation pattern of the Hsp 70 gene region in the infected susceptible snail (manuscript in preparation). These data combined with recent reports showing that schistosomes have the ability to orchestrate non- random spatial repositioning of gene loci that are actively transcribed in the susceptible snail, indicates that the *B. glabrata*-schistosome relationship is highly complex [35].

To conclude, breeding the resistant BS-90 stock at higher temperature changes their phenotype; these previously resistant snails exhibit susceptibility to infection. These new data corroborated previous findings of the differential expression of Hsp 70 between resistant and susceptible snails. The new findings suggested that resistance of BS-90 snails to *S. mansoni* may be a temperature-dependent trait. Finally, these findings have implications for the spread of schistosomiasis in the context of global warming.
